# Actin-binding protein profilin1 is an important determinant of cellular phosphoinositide control

**DOI:** 10.1016/j.jbc.2023.105583

**Published:** 2023-12-21

**Authors:** Morgan M.C. Ricci, Andrew Orenberg, Lee Ohayon, David Gau, Rachel C. Wills, Yongho Bae, Tuhin Das, David Koes, Gerald R.V. Hammond, Partha Roy

**Affiliations:** 1 Department of Cell Biology, University of Pittsburgh, Pittsburgh, Pennsylvania, USA; 2 Department of Bioengineering, University of Pittsburgh, Pittsburgh, Pennsylvania, USA; 3 Department of Pathology and Anatomical Science, University at Buffalo, Buffalo, New York, USA; 4 Tavotek Biotherapeutics, Spring House, Pennsylvania, USA; 5 Department of Computational and Systems Biology, University of Pittsburgh, Pittsburgh, Pennsylvania, USA; 6 Department of Pathology, University of Pittsburgh, Pittsburgh, Pennsylvania, USA

**Keywords:** profilin, phosphoinositides, PI(4,5)P_2_, PI(3,4,5)P_3_, PI(3,4)P_2_, SHIP2

## Abstract

Membrane polyphosphoinositides (PPIs) are lipid-signaling molecules that undergo metabolic turnover and influence a diverse range of cellular functions. PPIs regulate the activity and/or spatial localization of a number of actin-binding proteins (ABPs) through direct interactions; however, it is much less clear whether ABPs could also be an integral part in regulating PPI signaling. In this study, we show that ABP profilin1 (Pfn1) is an important molecular determinant of the cellular content of PI(4,5)P_2_ (the most abundant PPI in cells). In growth factor (EGF) stimulation setting, Pfn1 depletion does not impact PI(4,5)P_2_ hydrolysis but enhances plasma membrane (PM) enrichment of PPIs that are produced downstream of activated PI3-kinase, including PI(3,4,5)P_3_ and PI(3,4)P_2,_ the latter consistent with increased PM recruitment of SH2-containing inositol 5′ phosphatase (SHIP2) (a key enzyme for PI(3,4)P_2_ biosynthesis). Although Pfn1 binds to PPIs *in vitro*, our data suggest that Pfn1’s affinity to PPIs and PM presence in actual cells, if at all, is negligible, suggesting that Pfn1 is unlikely to directly compete with SHIP2 for binding to PM PPIs. Additionally, we provide evidence for Pfn1’s interaction with SHIP2 in cells and modulation of this interaction upon EGF stimulation, raising an alternative possibility of Pfn1 binding as a potential restrictive mechanism for PM recruitment of SHIP2. In conclusion, our findings challenge the dogma of Pfn1’s binding to PM by PPI interaction, uncover a previously unrecognized role of Pfn1 in PI(4,5)P_2_ homeostasis and provide a new mechanistic avenue of how an ABP could potentially impact PI3K signaling byproducts in cells through lipid phosphatase control.

Polyphosphoinositides (PPIs) constitute a class of minority phospholipid molecules that account for less than 5% of lipid content in eukaryotic cells (1). Despite their low abundance, PPIs are incredibly important functionally as these serve as lipid-signaling molecules influencing a diverse range of cellular functions, including actin cytoskeletal regulation, migration, proliferation, growth, metabolism, and differentiation (2, 3, 4). There are seven different molecular species of PPIs in cells including PI(3)P, PI(4)P, PI(5)P, PI(4,5)P_2_ (referred to as PIP_2_ from hereon), PI(3,5)P_2_, PI(3,4)P_2_, and PI(3,4,5)P_3_ (referred to as PIP_3_ from hereon). Metabolic conversion of one PPI to other forms in cells occurs by site-specific actions of various lipid kinases, phosphatases, and lipases. Dysregulated expressions and/or activities (through mutation) of PPI-modifying enzymes have links to a number of diseases, including cancer (5, 6).

PIP_2_, the most abundant PPI species in cells, is subjected to metabolic turnover when receptor tyrosine kinases (RTKs—such as epidermal growth factor receptor (EGFR) and platelet derived growth factor receptor (PDGFR)) or G protein–coupled receptors (GPCRs) are activated in response to appropriate ligands. The two major pathways of PIP_2_ turnover downstream of RTK activation are phospholipase-Cγ (PLCγ)-mediated hydrolysis of PIP_2_ into inositol triphosphate (IP_3_) and diacylglycerol (DAG), and class I PI3K–mediated conversion of PIP_2_ to PIP_3_ (7, 8). PIP_3_ can be further subjected to 5′-phosphatase–mediated dephosphorylation to generate PI(3,4)P_2_. The 5′-phosphatases belong to either SH2-containing inositol 5′ phosphatase (SHIP) (hematopoietic cell restricted SHIP1 and broadly expressed SHIP2) or inositol polyphosphate 5-phosphate (INPP5) (INPP-E, INPP-J, INPP-K) families (9, 10). PI(3,4)P_2_ can be also synthesized by an alternative pathway that involves sequential phosphorylation of PI at the 4′-OH position of the myo-inositol ring of PI, followed by one at the 3′-OH position by the action of class II PI3K (11). However, the vast majority of PI(3,4)P_2_ is synthesized through SHIP2-mediated hydrolysis of class I PI3K–produced PIP_3_ (12), a finding that is consistent with the synthesis of PI(3,4)P_2_ lagging that of PIP_3_ in cells (13). PI3K-generated PPIs are downregulated primarily by the action of two lipid phosphatases, namely phosphatase and tensin homolog deleted on chromosome 10 (PTEN—hydrolyzes the 3′-phosphate of PIP_3_ and PI(3,4)P_2_ to generate PIP_2_ and PI(4)P, respectively) and INPP4-A/B, hydrolyzes the 4′-phosphate of PI(3,4)P_2_ to generate PI(3)P) (12, 14, 15).

PPIs regulate the activity and/or spatial localization of a number of actin-binding proteins (ABPs) through direct interactions. ABPs generally interact with PPIs through multivalent electrostatic interactions, but the strength of the interaction varies drastically depending on the type of ABP (16). While PIP_2_ has been shown to activate ABPs that enhance actin assembly (such as N-WASP, WAVE) (17), much less is known whether actin-regulatory proteins themselves could also be an integral part in regulating PPI signaling at the plasma membrane (PM).

In this study, we investigated the role of profilin1 (Pfn1), the founding member and ubiquitously expressed member of profilin family of ABPs in cellular regulation of PPIs. Pfn1 is a small actin monomer-binding protein that plays crucial role in actin assembly and various actin-dependent biological processes (18). Apart from binding to actin and a repertoire of poly-proline domain-bearing proteins, Pfn1 is also capable of interacting with various PPIs. At least in an *in vitro* setting, Pfn1 binds to PIP_2_, PIP_3_, and PI(3,4)P_2_ lipid micelles (19) and has a slightly higher affinity for PI(3,4)P_2_ relative to the other two PPIs (20). Although Pfn1 binds to PIP_2_-reconsituted giant unilamellar vesicles (GUVs) (16, 21), a direct experimental evidence for Pfn1’s binding to any specific PM PPI species in actual cells is still lacking. Structural overlap between the actin- and the PPI-binding sites of Pfn1 explains the mutual exclusivity of Pfn1’s interactions with these ligands in biochemical assays. Based on these findings, it was proposed that that PPI binding could be a potential mechanism to negatively regulate Pfn1’s interaction with actin (22). In a previous study, we performed knockdown-rescue experiments to demonstrate that Pfn1 depletion in MDA-MB-231 (referred to as MDA-231 hereon) breast cancer cells prominently enhances PM accumulation of PI(3,4)P_2_ downstream of activated RTK (EGFR and PDGFR) without impacting RTK activation (23). These data suggested that Pfn1 could have a role in PPI signaling regulation at the PM in cells. Whether Pfn1’s inhibitory effect on PI(3,4)P_2_ is also true for normal (*i.e.*, nontumorigenic) cells and the underlying mechanisms remained unclear. The present study shows for the first time that perturbing Pfn1 expression has a major consequence on cellular PIP_2_ content. Although Pfn1 has no discernible impact on PLCγ-mediated PIP_2_ hydrolysis pathway, Pfn1 acts as a brake on PI3K-mediated PIP_2_ turnover pathway in cells upon EGF stimulation. We further identify a novel interaction between Pfn1 with SHIP2, and demonstrate Pfn1-dependent alteration of PM recruitment of SHIP2 which, together with changes in PIP_3_, to potentially explain how Pfn1 may inhibit PI(3,4)P_2_ biosynthesis in cells.

## Results

### Pfn1 depletion stimulates biosynthesis of PI(3,4)P_2_

**Figure 1:**
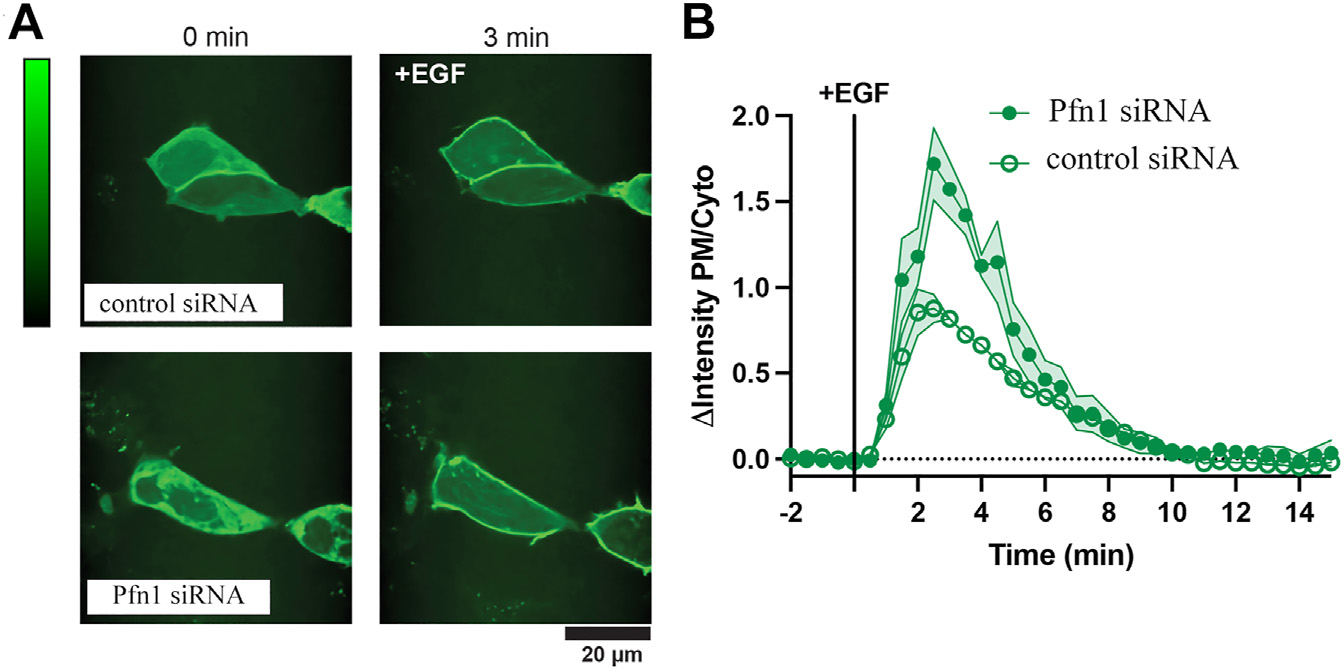
**Pfn1 knockdown increases PI(3,4)P**_**2**_**production at the PM**. *A*, representative confocal images of cPHx3 expressing control *versus* Pfn1 knockdown HEKE-293 cells pre-EGF and ∼3 min post-EGF stimulation. *B*, relative kinetics of PM recruitment of cPHx3 (represented as a ratio of PM-to-cytoplasmic intensity and normalized to those values averaged over five pre-EGF stimulation frames) of the two groups of transfected cells. Spread of the data represent ± SEM based on measurements of 23 to 26 number of cells pooled from three experiments. PM, plasma membrane; Pfn1, profilin1.

PPIs including PI(3,4)P_2_ are under dynamic control of PM-recruited lipid kinases and phosphatases. Our previous finding of PM enrichment of PI(3,4)P_2_ induced by the loss of Pfn1 expression was strictly based on the results of immunostaining experiments limited to a single breast cancer cell line at a fixed timepoint after growth factor (EGF or PDGF) stimulation (23). Therefore, it remained unclear whether: a) Pfn1 impacts synthesis *versus* turnover of PI(3,4)P_2_ and b) Pfn1’s inhibitory effect on PI(3,4)P_2_ is also true in normal (nontumorigenic) cells. To address these gaps, we performed live imaging of a high-avidity PI(3,4)P_2_-specific biosensor and monitored the kinetics of EGF-induced PM content of PI(3,4)P_2_ with or without acute Pfn1 depletion by siRNA transfection in HEK-293, a normal human embryonic kidney epithelial cell line. The PI(3,4)P_2_ biosensor utilizes a tandem trimer of the C-terminal pleckstrin-homology (PH) domain of human TAPP1 (referred to as cPHx3-TAPP1, which binds selectively to PI(3,4)P_2_) that is fused to EGFP and a nuclear export signal sequence, as previously described by the Hammond group (12). Immunoblot shown in Fig. S1 confirms Pfn1 downregulation in HEK-293 cells, following transfection of smart-pool siRNA (distinct from a single-target siRNA sequence used in our previous study (23)). For both nontargeting control- and Pfn1-siRNA transfected cells, the PM fluorescence of cPHx3-TAPP1 (normalized to its cytoplasmic fluorescence to account for cell-to-cell variability in the expression of the biosensor) peaked sometime within 2 to 3 min after EGF stimulation. However, the peak amplitude of increase in cPHx3-TAPP1 fluorescence at the PM was higher in Pfn1 knockdown relative to control cells (Fig. 1, *A* and *B*). Within 10 min, the PM fluorescence of cPHx3-TAPP1 reduced to its baseline level in either transfected cell type, and Pfn1 knockdown cells, if at all, displayed a faster decline of the PM fluorescence of cPHx3-TAPP1 to its baseline level *versus* control cells. This data demonstrates that Pfn1 depletion leads to PM enrichment of PI(3,4)P_2_ primarily by boosting its synthesis rather than slowing down its turnover and that Pfn1-dependent alteration in PI(3,4)P_2_ is also applicable to normal cells.

### Pfn1 is an important molecular determinant of cellular PI(4,5)P_2_ content

**Figure 2:**
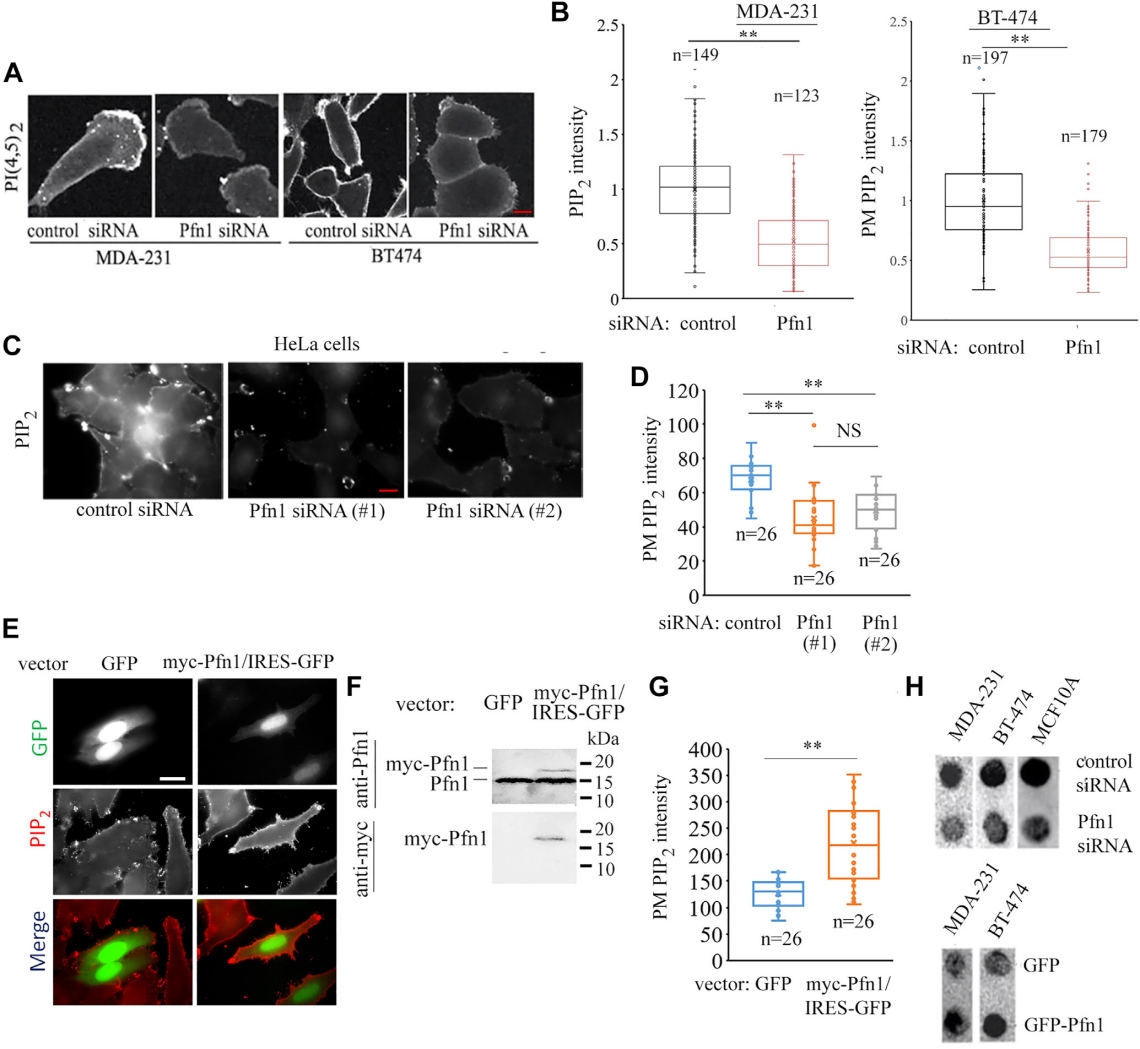
**Pfn1 is a major regulator of cellular PIP**_**2**_**content.***A* and *B*, representative images (*panel A*; the scale bar represents −10 μm) and quantification (*panel B*; n indicates cell number pooled from two experiments) of the overall staining intensity of PIP_2_ with or without Pfn1 knockdown in MDA-231 and BT-474 cells. *C* and *D*, representative images (*panel C*; the scale bar represents −10 μm) and quantification (*panel D*; n indicates cell number pooled from two experiments) of PM staining intensity of PIP_2_ with or without Pfn1 knockdown by two different siRNAs (siRNA #1—single-target; siRNA #2—smart pool) in HeLa cells. *E*–*G*, representative images (*panel E*; the scale bar represents −10 μm) and quantification (*panel G*; n indicates cell number pooled from two experiments) of PM staining intensity of PIP_2_ in GFP+ cells transfected with plasmids encoding myc-Pfn1 (along with IRES-GFP) or IRES-GFP as control. Immunoblot of TCL with anti-myc antibody shown in *panel F* confirms myc-Pfn1 overexpression. *H*, lipid dot blot images showing the effects of either silencing or overexpression of Pfn1 on total cellular PIP_2_ level in the indicated cell lines (GFP expressing subline serves as the control for GFP-Pfn1–overexpressing cells). IRES, internal ribosome entry site; Pfn1, profilin1; PM, plasma membrane; TCL, total cell lysate.

Since sequential turnover of PIP_2_ gives rise to the production of PIP_3_ and subsequently PI(3,4)P_2_ in cells, we asked whether altering Pfn1 expression causes any major imbalance in cellular PIP_2_ content. To address this question, we utilized immunostaining and lipid dot-blot assay to measure Pfn1-dependent changes in PIP_2_ in a number of different cell types. First, our PIP_2_ immunostaining experiments revealed that silencing Pfn1 expression in human breast cancer (MDA-MB-231 [referred to as MDA-231 hereon] and BT-474) cell lines leads to a significant decrease in the PIP_2_ content (Figs. 2, *A* and *B* and S1 confirms siRNA-mediated knockdown of Pfn1 expression in these two cell lines). We also verified these results in cervical cancer HeLa cell line with the use of two different Pfn1 siRNAs (Fig. 2, *C* and *D*; knockdown of Pfn1 expression in HeLa cells is shown in Fig. S1). As a complementary experiment, we transfected HeLa cells with an expression vector encoding myc-Pfn1 (coexpressed with GFP reporter through an internal ribosome entry site arrangement) with internal ribosome entry site-GFP backbone vector transfection serving as control and assessed PM PIP_2_ staining intensity in GFP-positive cells in the two groups. Consistent with the knockdown data, acute Pfn1 elevation through overexpression of myc-Pfn1 led to increased PM PIP_2_ content in HeLa cells (Fig. 2, *E*–*G*). Note that although PIP-kinase–mediated phosphorylation of PI(4)P is a major route for biosynthesis of PIP_2_, PI(4)P content, if at all, was negligibly increased in HeLa cells when Pfn1 expression was silenced, therefore suggesting that PIP_2_ depletion upon loss of Pfn1 expression is not due to limited substrate availability of PI(4)P (Fig. S2). Next, to determine the effect of Pfn1 perturbation on the total cellular PIP_2_ pool, we performed biochemical PIP_2_ lipid dot-blot assay with lipids extracted from both normal (MCF10A, a nontumorigenic immortalized mammary epithelial cell line) and breast cancer cell lines (MDA-231, BT-474)—all three cell lines showed reduced total PIP_2_ content when Pfn1 expression was silenced (Fig. 2*H*; knockdown of Pfn1 expression in MCF10A cells is shown in Fig. S1). Consistent with these knockdown data, dot-blot assays performed in Pfn1 overexpression settings revealed higher total PIP_2_ content in GFP-Pfn1 overexpressing sublines of both MDA-231 and BT474 cells *versus* their respective GFP subline controls (Fig. 2*K*). Based on these immunostaining and biochemical data revealing Pfn1′s ability to positively impact the total and PM content of PIP_2_, we conclude that Pfn1 is an important molecular determinant of cellular PIP_2_ levels and that Pfn1-dependent regulation of PIP_2_ abundance in a generalizable feature across different cell types.

### Pfn1 depletion does not affect PLC-mediated PIP_2_ hydrolysis but enhances PIP_3_ generation in cells

**Figure 3:**
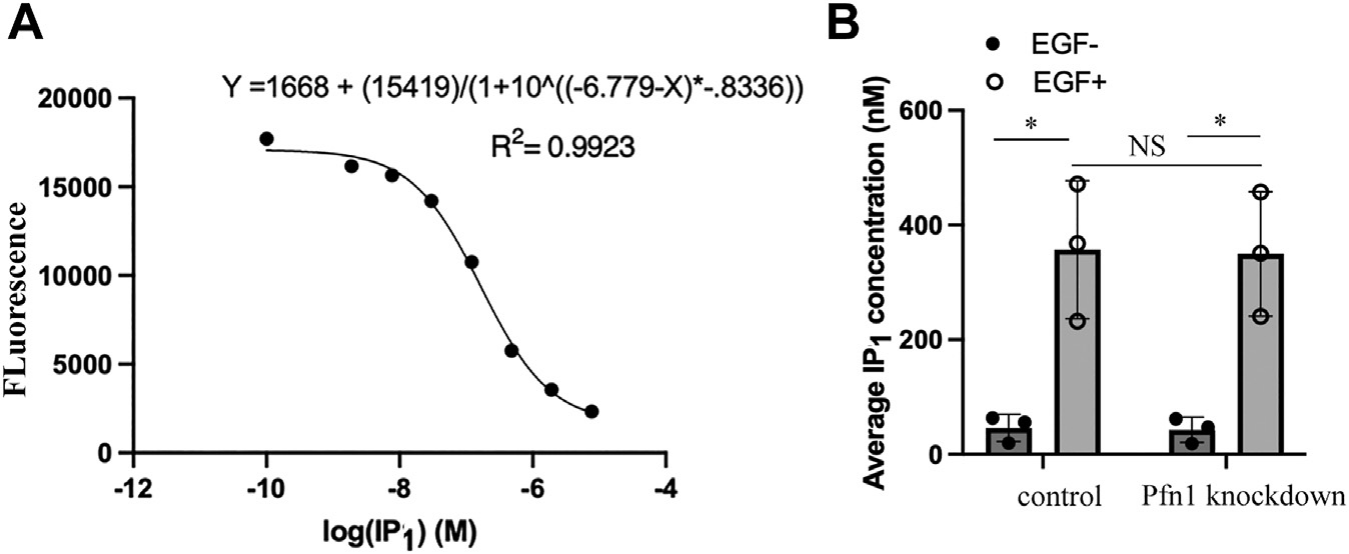
**Loss of Pfn1 does not alter EGF-induced PIP_2_ hydrolysis in cells**. Average IP_1_ concentration in control *versus* Pfn1 knockdown MDA-231 cells before and 30 min after EGF stimulation (*panel B*); *panel A* shows the standard curve for the FRET-based IP_1_ quantification assay. These data are summarized from three individual experiments (∗*p* < 0.05; NS, not significant). Pfn1, profilin1; IP_1_, inositol monophosphate.

**Figure 4:**
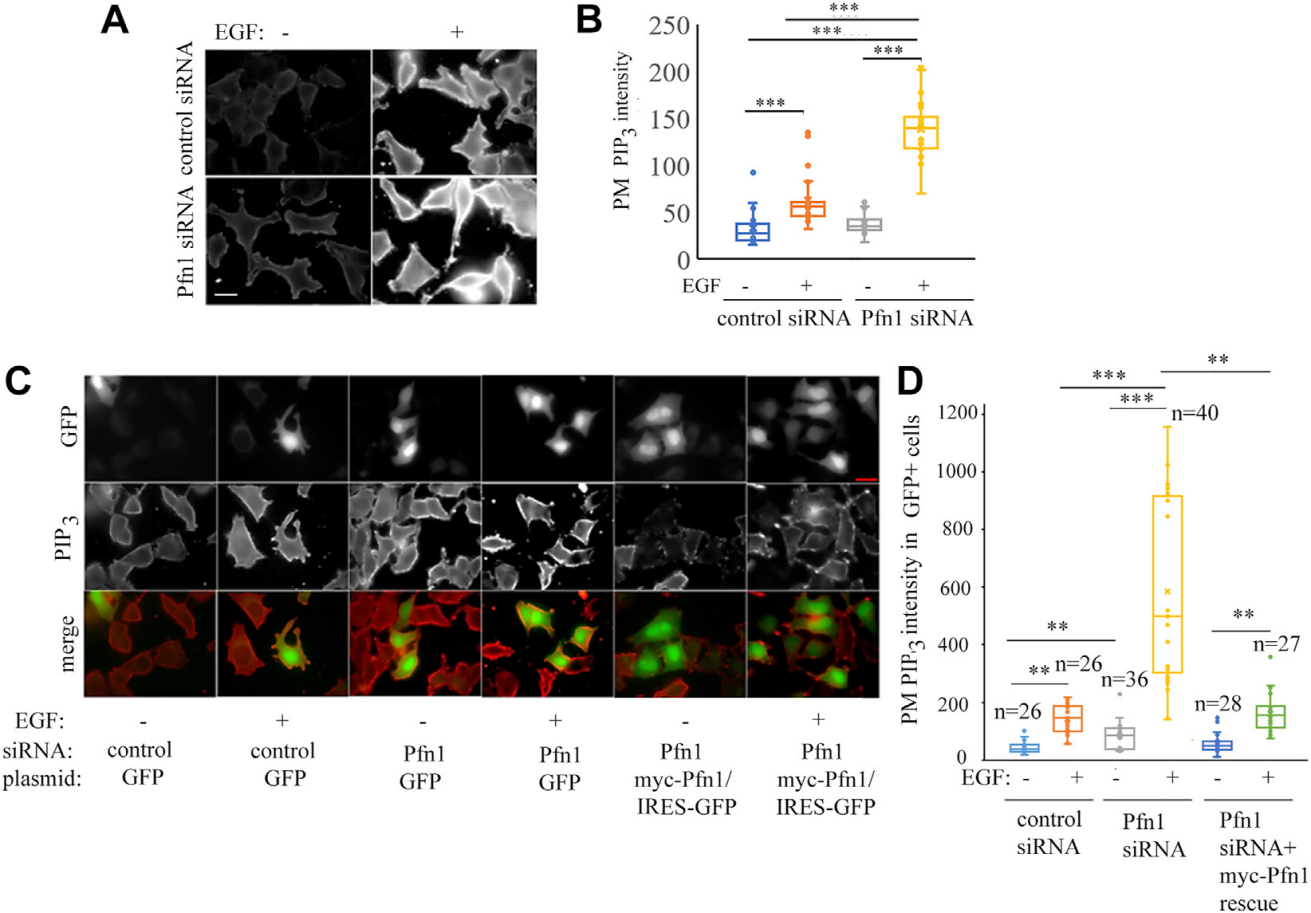
**Pfn1 acts as a brake on PIP**_**3**_**generation.***A* and *B*, representative images (*panel A*; the scale bar represents −20 μm) and quantification (*panel B*) of PM staining intensity of PIP_3_ in control *versus* Pfn1 knockdown HeLa cells before and 5 min after EGF stimulation. *C* and *D*, representative images (*panel C*; the scale bar represents −20 μm) and quantification (*panel D*) of PM staining intensity of PIP_3_ in GFP+ HeLa cells transfected with the indicated siRNAs and plasmids before and 5 min after EGF stimulation (∼50 cells were from randomly chosen 60× fields pooled from three experiments were analyzed) [∗∗∗*p* < 0.001; ∗∗*p* < 0.01] (the scale bar represents –10 μm).Pfn1, profilin1; PM, plasma membrane.

Activation of EGFR triggers two major PIP_2_ turnover pathways: PLC-mediated hydrolysis of PIP_2_ to IP_3_ and DAG, and PI3K-mediated conversion of PIP_2_ to PIP_3_. Next, to determine whether Pfn1 depletion has any effect on PLC pathway, we performed a competitive FRET-based biochemical assay to measure IP_1_ (a rapid degradation product of IP_3_ that serves as a biochemical readout of PLC pathway) content in control *versus* Pfn1 knockdown MDA-231 cells with or without EGF stimulation. As expected, EGF stimulation leads to a dramatic increase in cellular IP_1_ level regardless of the Pfn1 expression status, but we did not see any significant difference in the fold change in EGF-induced IP_1_ accumulation between the two groups of cells (Fig. 3). These data suggest that Pfn1 does not play any discernible role in modulating PLC-mediated PIP_2_ hydrolysis pathway. Contrasting these findings, EGF-induced PM accumulation of PIP_3_ was found to be significantly enhanced in both MDA-231 (Fig. S3) and HeLa cells when Pfn1 expression was silenced (Fig. 4*A*), a trend that was reversed upon re-expression of Pfn1 as demonstrated in knockdown-rescue experiments performed in HeLa cells (Fig. 4*B*). These data support a model that Pfn1 acts as a brake on PI3K-mediated turnover of PIP_2_.

### Pfn1 negatively regulates PM recruitment of SHIP2

**Figure 5:**
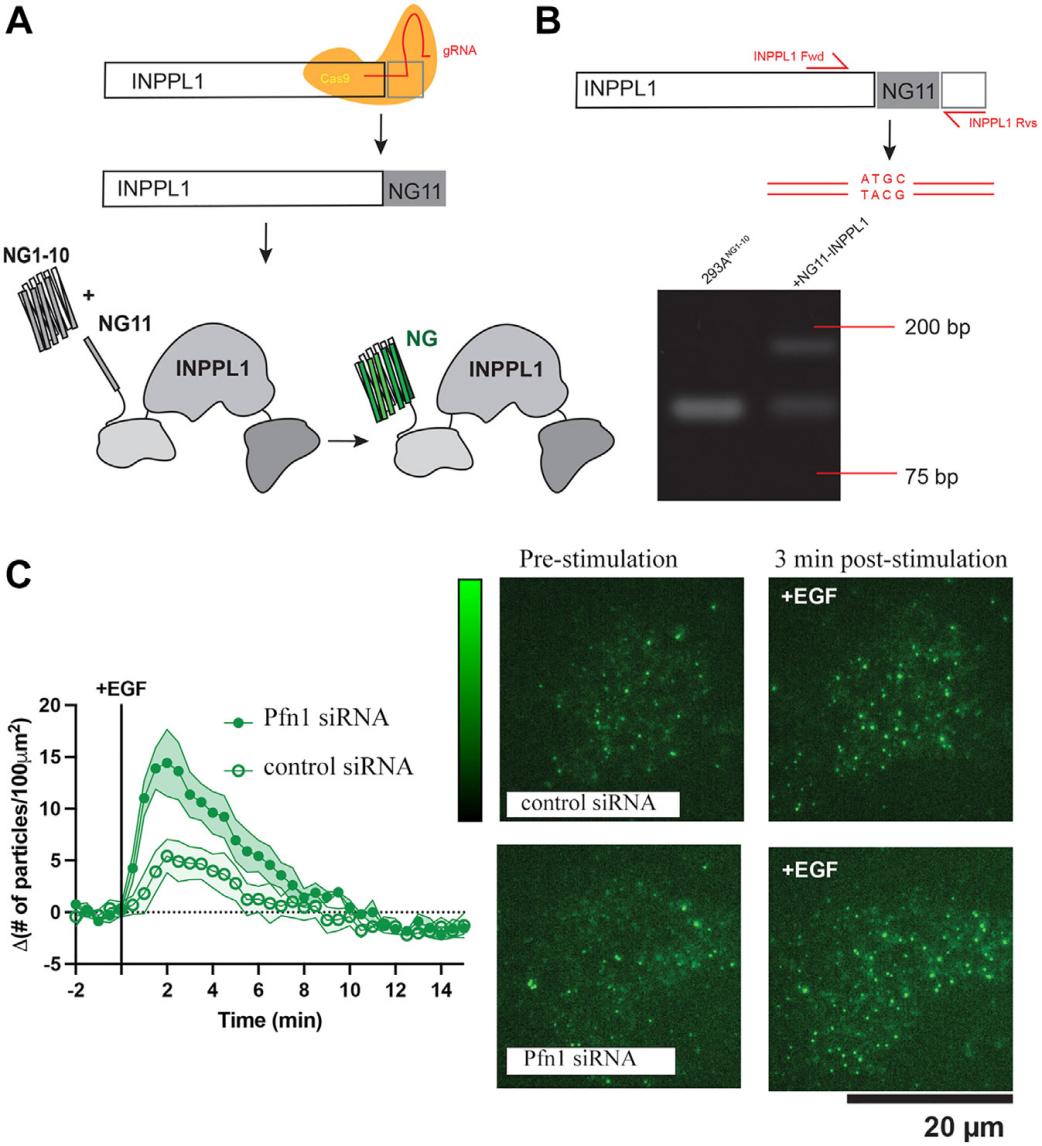
**Pfn1 depletion enhances PM recruitment of SHIP2.***A*, CRISPR-Cas9 genomic editing strategy of INPPL1 (SHIP2): a guide RNA–Cas9 protein complex and a single-stranded homology-directed repair (HDR) template is electroporated into HEK293A cells stably expressing NG1-10. Using the split fluorescent protein approach, this tags the SHIP2 protein with the 11th strand of the *Neon Green* (NG) beta barrel that assembles with the NG1-10 to form a working fluorescent tag. *B*, genomic confirmation of INPPL1 tagging. INPPL1-specific forward (Fwd) and reverse (Rvs) primers amplified NG11 carboxy-terminal knock-in region of the gene. *C*, representative TIRF microscopy images of 293A-NG2-1-10:INPPL1-NG11 pre-EGF and ∼3 min post-EGF stimulation. Single-particle PM recruitment measured by number of particles in defined region of interest (ROI) of 100 μm^2^ area (particle density). The particle density at baseline (five frames prestimulation) is subtracted to yield the change in density upon EGF stimulation. Three to four experimental replicates (34–35 cells total for each condition) presented as grand means ± SEM. INPP1, inositol polyphosphate 1-phosphate; Pfn1, profilin1; PM, plasma membrane; SHIP, SH2-containing inositol 5′ phosphatase; TIRF, total internal reflection fluorescence.

In nonhematopoietic cells, SHIP2’s action is primarily responsible for biosynthesis of PI(3,4)P_2_ from its precursor PIP_3_, while 3′- and 4′- inositol phosphatases, namely PTEN and INPP4B, catalyze P(3,4)P_2_ turnover. Through immunoblot analyses of extracts prepared from various cell lines, we confirmed that Pfn1 depletion does not affect the expressions of SHIP2, PTEN, INPP4 as well as C-terminal phosphorylation status of PTEN (pS380/pT382/pT383, which maintains PTEN in an inactive form (24)) (Fig. S4). These data prompted us to examine whether loss of Pfn1 has any effect on PM localization of SHIP2 (a requirement for SHIP2’s lipid phosphatase activity) in cells. To address this question, we applied the CRISPR-Cas9 gene editing method to endogenously tag the INPPL1 (SHIP2) with a fluorescent protein. We followed the split fluorescent protein approach by integrating the 11th beta strand of Neon green (NG) to the INPPL1 gene in our stably expressing HEK293A NG1-10 cell line (referred to as 293A^NG1-10^) (25, 26) (Fig. 5*A*). By tagging the C terminus of INPPL1 with NG11, NG1-10 is able to self-complement with the final beta barrel component and form a functional fluorescent NG tag at our gene of interest (25, 26). We then confirmed successful integration of NG11 in INPPL1 through PCR genotyping (Fig. 5*B*). Forward and reverse primers were designed that specifically adhered to INPPL1 directly upstream and downstream of the edited site. The resulting PCR products showed 119 bp INPPL1 amplified region without genomic integration and a 176 bp INPPL1 amplified region with NG11 genomic integration (Fig. 5*B*). We then measured PM recruitment of SHIP2 in our NG endogenously tagged cell line through single molecule imaging with total internal reflection fluorescence (TIRF) microscopy. We tracked SHIP2 recruitment to the PM in response to EGF stimulation with or without Pfn1 knockdown. Our experiments showed that EGF stimulation increased SHIP2 recruitment to the PM, a feature that was further enhanced when Pfn1 expression was knocked down (Fig. 5*C*). These data demonstrate that loss of Pfn1 expression promotes EGF-induced PM recruitment of SHIP2.

**Figure 6:**
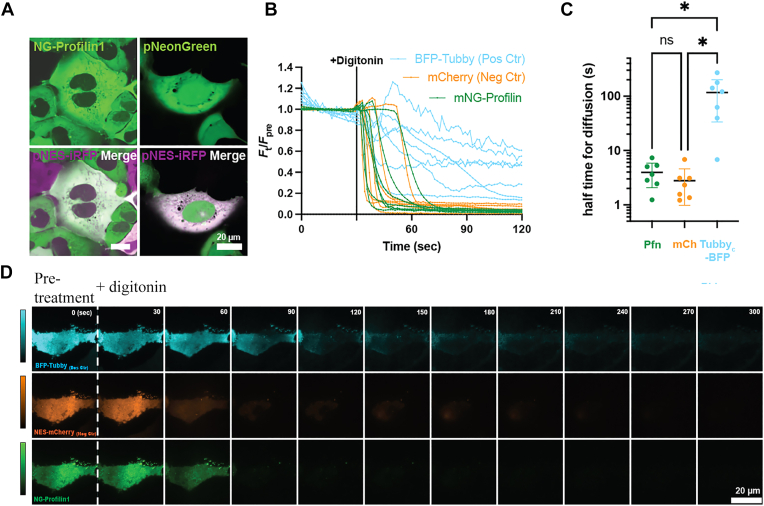
**Pfn1 does not have discernible PM localization in cells.***A*, representative confocal images of endogenous Pfn1 cytosolic localization in 293A-NG2-1-10:NG11-Pfn1 cells (pNES-iRFP-C1 was expressed as cytosolic marker and pNeonGreen-C1 was used as cytosolic control). *B–D*, *panal B* shows quantification of endogenous Pfn1, mCherry, and BFP-Tubby_c_ residence in 293A-NG2-1-10:NG11-Pfn1 cells, following cell permeabilization (pmCherry-C1 was expressed as a negative-cytosolic control and pTag2BFP-Tubby_c_ was expressed as a positive-PM binding control). Cells were permeabilized with digitonin and the fluorescence intensity was measured over time in the 405, 488, and 640 channels and baseline corrected to the first 30 s baseline (prior to digitonin treatment) in respective channels. Each line represents an individual cell from the three experimental repeats. *Panel C* shows the half-time for diffusion determined by the length of time in seconds until the fluorescence intensity reduced by half, following cell permeabilization. Data points represent individual cells taken from three experimental repeats (2–3 cells/repeat; ∗*p* < 0.05) *Panel D* shows the representative TIRF microscopy image montage of endogenous Pfn1, negative-cytoplasmic control mCherry, and positive-PM binding control BFP-Tubby_c_ PM residence, following cell permeabilization in HEK293A-NG2-1-10:NG11-Pfn1 cells. iRFP, near infrared fluorescent protein; Pfn1, profilin1; PM, plasma membrane; TIRF, total internal reflection fluorescence.

Since Pfn1 is known to bind to various PPI micelles, we initially considered a possibility that Pfn1 and SHIP2 may compete for PPI binding at the PM, and therefore an absence of Pfn1 would augment PM recruitment of SHIP2 through relief of this competitive inhibition. However, the validity of this postulate requires a direct demonstration of the PM targeting capability of Pfn1 in a PPI-dependent manner in cells, which has not been demonstrated to date. To explore this scenario, we applied the same split fluorescent protein technique demonstrated above, and knocked in NG11 at the endogenous locus of Pfn1 in our stably expressing 293A^NG1-10^ cells. The homology-directed repair template (HDRT) and guide RNA (gRNA) sequences were obtained from the OpenCell database. Using our NG-Pfn1 cell line, we produced confocal representative images that confirmed predominantly cytosolic localization of mNG-Pfn1, reflective of our cytosolic control pNG-C1 (Fig. 6*A*), similar to the localization of endogenous Pfn1 reported in other studies (27, 28). We then performed time-lapse TIRF microscopy of mNG-Pfn1 cells to examine mNG-Pfn1 content at the PM, following depletion of its cytosolic content by digitonin-mediated cell permeabilization. We also expressed pmCherry-C1 as a negative-cytosolic control (which is expected to rapidly leave the cell upon permeabilization), and pTag2BFP-Tubby(c)-R332H (binds PIP_2_ at the PM) as a positive PM-binding control that is expected to remain within the cell for longer periods following permeabilization. We measured the fluorescent intensity of each of these three constructs before and after cell permeabilization, and normalized the post-permeabilization intensity values relative to the respective average baseline values which were calculated over a period of 30 s prior to digitonin treatment. As shown in Figure 6, *B* and *D*, both pmCherry-C1 and mNG-Pfn1 rapidly exist the cell upon permeabilization and have a much shorter residence time in the cell relative pTag2BFP-Tubby(c)-R332H as measured by the half-time for fluorescence decay (Fig. 6*C*). These data suggest that Pfn1’s binding to the PM in cells, if at all, is negligible.

**Figure 7:**
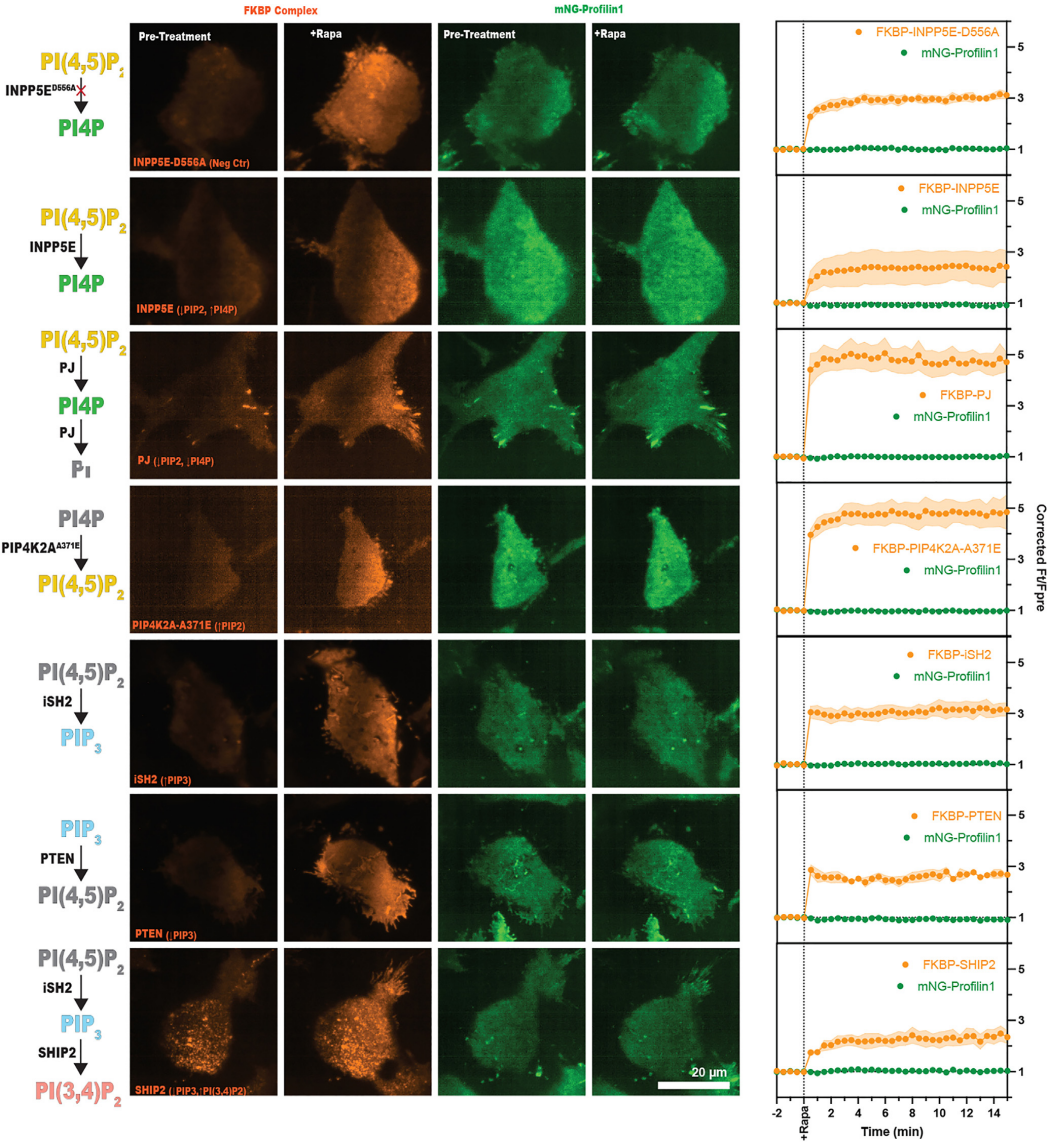
**Pfn1 does not have discernible affinity for PM PPIs in cells**. FKBP-FRB chemical dimerization system strategy was used to drive several lipid phosphatases and kinases to the PM and induce the enrichment or loss of different phosphoinositide species. FRB was fused to a Lyn11 sequence and localized to the PM, FKBP was fused to the different enzymes and recruited to the PM, following rapamycin stimulation. Rapamycin dimerizes the FRB and FKBP units and exposes the enzymes to their membrane substrates to induce catalytic activity. mNG-Pfn1 fluorescence intensity at the PM was measured pre and postrapamycin stimulation to determine whether the enrichment or loss of different lipids altered the proteins presence on the membrane. Grand means from three to four experimental repeats (26–37 cells total; 6–11 cells/repeat) were produced and show fluorescence intensity of the FKBP subunit and mNG-profilin1. Bars represent ±s.e.m. FKBP, FK506-binding protein; FRB, FKBP-rapamycin binding; Pfn1, profilin1; PM, plasma membrane; PPI, polyphosphoinositide.

To reinforce our foregoing observation, we wanted to further examine whether artificial enrichment or loss of any specific PPI species at the PM using the FK506-binding protein–rapamycin binding (FRB) chemical dimerization technique is sufficient to recruit Pfn1 to the PM. The Lyn11 sequence fused to the FRB domain of mammalian target of rapamycin kinase unit localizes the structure to the PM and addition of rapamycin induces dimerization with the FK506-binding protein unit fused to specific lipid kinases or phosphatases (29), this allows acute recruitment and activity of the enzymes at the membrane. Using this approach, we enhanced PM recruitment of a variety of lipid modifying enzymes in HEK-293 cells endogenously tagged with mNG-Pfn1. These enzymes include: INPP5E-D556A (a catalytically dead mutant of INPPE that blocks PIP_2_’s conversion to PI4P), INPPE (promotes PIP_2_’s conversion to PI4P), pseudojanin, a fusion of Sac and INPP5E phosphatase, that promotes dephosphorylation of PIP_2_ to PI, PIP4K2A-A371E (a mutant form of PIP4K2A that changes its function from 4-kinase to 5-kinase facilitating conversion of PI4P to PIP_2_), inter-src homology domain from the regulatory p85 subunit of PI3K to recruit catalytic p110 subunit and activate class I PI3K promoting PIP_2_’s conversion to PIP_3_, PTEN (dephosphorylates both PIP3 and PI(3,4)P_2_), and SHIP2 (stimulates PI(3,4)P_2_ generation). Interestingly, PM recruitment of none of these PPI-modifying enzymes was able to recruit mNG-Pfn1 at the PM (Fig. 7). These data suggest that Pfn1’s affinity to PM PPI in actual cells, if at all, is negligible. Based on these observations, we conclude that it is highly unlikely that Pfn1 and SHIP2 directly compete for PPI binding at the PM, and therefore, enhanced PM recruitment of SHIP2 upon Pfn1 depletion might involve alternate mechanism.

**Figure 8:**
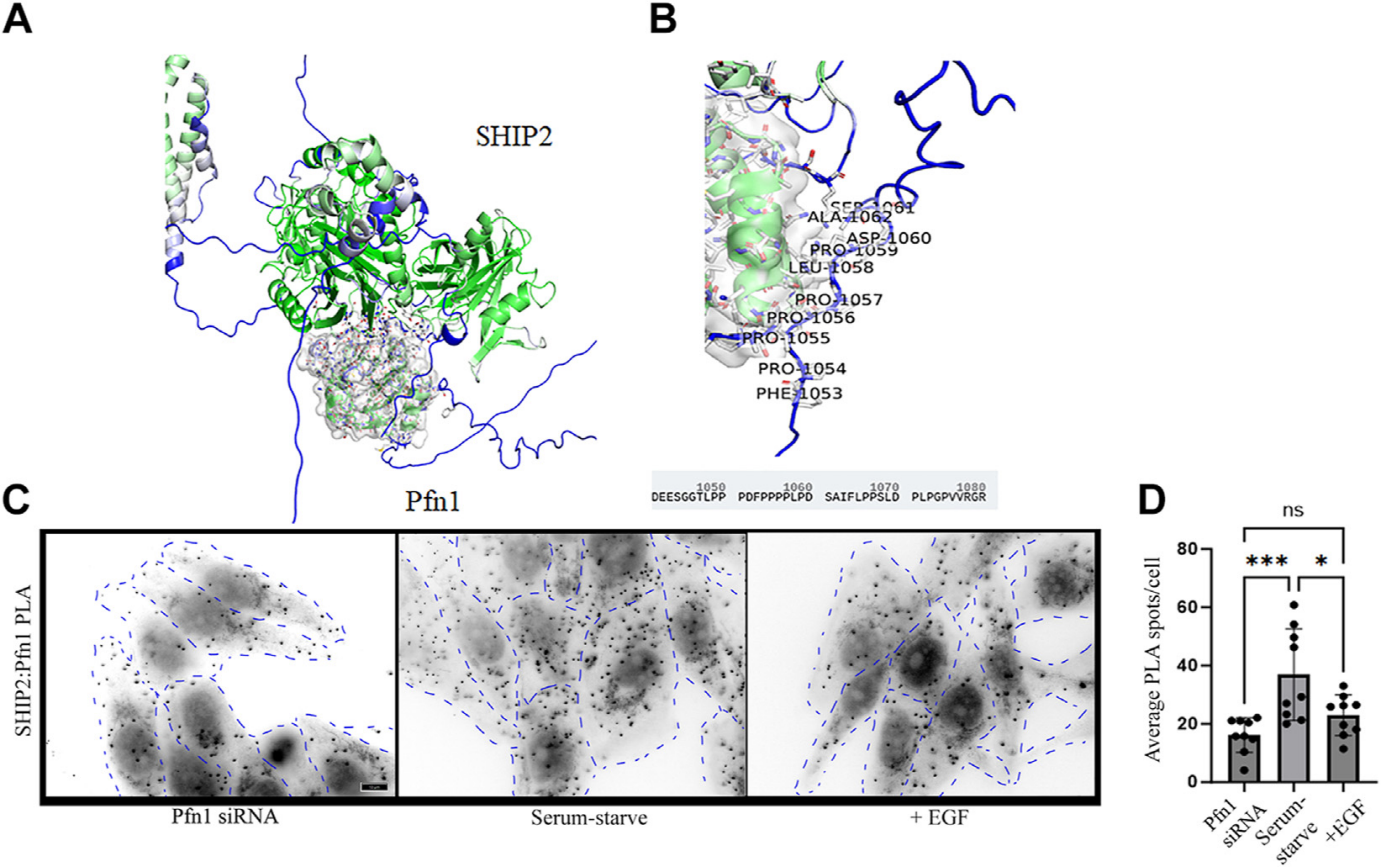
**Evidence for Pfn1–SHIP2 interaction in cells**. *A* and *B*, highest-ranked alpha-fold prediction of Pfn1’s interface with SHIP2 at its C-terminal polyproline-rich domain and the involved amino acid sequence for binding to Pfn1. *C* and *D*, representative images (*panel C*; the scale bar represents −10 μm) of proximity ligation assay to demonstrate endogenous Pfn1–SHIP2 interaction in MDA-231 cells with or without EGF stimulation (fluorescence images were intensity inverted for visual clarity of the PLA spots; *dotted lines* outline the cell periphery). As control, cells were transfected with Pfn1 siRNA (show a pronounced decrease in PLA spots) to demonstrate the specificity of interaction. *Panel D* quantifies EGF-induced changes in the average number of Pfn1:SHIP2 PLA spots/cell. Data are summarized from image analyses of five random 60× fields/experiment pooled from two independent experiments. Pfn1, profilin1; SHIP, SH2-containing inositol 5′ phosphatase.

Interestingly, when we performed a query of OpenCell database (https://opencell.czbiohub.org/) (30), we found SHIP2 as a potential interacting partner of Pfn1 (these data were based on mass spectrometry–based identification of peptide fragments of SHIP2 in immunoprecipitated endogenously tagged fluorescent Pfn1). Our unpublished Yeast two-hybrid screening also detected Pfn1’s binding to coding sequence of SHIP2 with moderate confidence. Furthermore, our Alpha-fold analyses predicted Pfn1’s binding to SHIP2, involving polyproline-binding region of Pfn1 and proline-rich region located at the C terminal of SHIP2 (Fig. 8, *A* and *B*). Given these ancillary findings, we initially sought to examine Pfn1:SHIP2 binding by coimmunoprecipitation experiments in HEK-293 cells in the settings of overexpression of either myc-tagged Pfn1 or flag-tagged SHIP2. Unfortunately, we failed to detect Pfn1:SHIP2 binding by conventional immunoprecipitation, followed by immunoblot assays (data not shown). We speculate that this could be due to low abundance and/or low-affinity interaction of Pfn1:SHIP2 complex that is below the limit of detection by immunoblot analyses. However, by proximity ligation assay (PLA), we were able to detect endogenous Pfn1–SHIP2 interaction in MDA-231 cells and show a reduction in the average number of PLA spots/cell when cells were stimulated by EGF (Fig. 8, *C* and *D*), further suggesting that Pfn1:SHIP2 interaction is modulated by growth factor signaling.

## Discussion

This study reports several major novel findings that have broad significance. Firstly, our studies demonstrate for the first time that perturbing Pfn1 expression has major consequence on cellular PIP_2_ levels. Given PIP_2_’s central role in a repertoire of biological processes ranging from regulation of ABPs to control of adhesion dynamics, ion channels, and intracellular trafficking (7), our findings open a new possibility of Pfn1-dependent changes in cellular phenotypes through additional regulation of PIP_2_ that extends beyond its direct effect on actin polymerization. Secondly, based on Pfn1’s ability to bind to PPI micelles and PIP_2_-receonstituted GUVs (16, 21), it has been speculated that a small pool of intracellular pool of Pfn1 may reside at the PM through specific PPI interaction, but never experimentally verified in actual cells. In this study, we engineered cells to endogenously tag Pfn1 with a fluorescent mNG reporter and demonstrate for the first time that not only mNG-Pfn1 exits cells rapidly upon permeabilization and at a similar rate as a pure cytosolic protein, acute PM recruitment of PPI-modifying kinases, and phosphatases (strategies that are well established for enriching PM for various PPIs including PIP_2_, PIP_3_, and PI(3,4)P_2_ (12, 31, 32, 33)), fail to bring mNG-Pfn1 to the PM in cells. These data suggest that Pfn1’s affinity for PM PPI in cells, if at all, is negligible, challenging the dogma of Pfn1’s binding to PM through PPI interaction. In fact, an earlier immunolocalization study performed in *Acanthamoeba Catellanii* predicted that between the two key isoforms of Pfn, Pfn2 is much more likely to localize to PM than Pfn1 (27). Although GUVs geometrically mimic PM of actual cells, reconstitution studies involving PIP_2_-enrichment GUV and purified Pfn1 fail to represent the scenario of cellular presence of many other lipid-binding proteins that have stronger affinity for PIP_2_ than Pfn1 and therefore can potentially compete Pfn1 out from PPI-binding. Furthermore, a biophysical study has shown that adding Pfn1 to PIP_2_-containing GUVs induces clustering of both Pfn1 and PIP_2_, resulting in destabilization and deformation of the GUV membrane (21). Therefore, Pfn1’s binding to PM PIP_2_ may also be functionally disadvantageous. Thirdly, we show that loss of Pfn1 does not alter EGF-induced PIP_2_ hydrolysis (a PLCγ-driven process) but stimulates production of PIP_3_ and PI(3,4)P_2,_ suggesting that between the two major metabolic turnover pathways of PIP_2_ downstream of activated EGFR, Pfn1 selectively acts as a brake on PI3K pathway without affecting PLCγ′s action. Fourthly, in the context of PI(3,4)P_2_ synthesis, we demonstrate that loss of Pfn1 expression enhances EGF-induced stimulation of PM recruitment of SHIP2, producing the first evidence for Pfn1-dependent control of PI3K-signaling byproduct through cellular localization of a lipid phosphatase.

Our studies show that Pfn1 positively regulates PIP_2_ without affecting the PI(4)P, the major precursor PPI for PIP_2_. One possible interpretation of this finding is that Pfn1 likely promotes metabolic conversion of PI(4)P to PIP_2_. PIP5K is the major kinase that is responsible for the conversion of PI4P to PIP5K. Rho family GTPases (RhoA, Rac1, and Cdc42), when activated, directly binds to and stimulates PIP5K activity (34). A previous study had shown loss of Pfn1 expression leads to downregulation of RhoA activation in chondrocytes (35). Therefore, Pfn1-dependent modulation of RhoA activity, if generalizable across cell types, could be a potential mechanism linking perturbation of Pfn1 expression and PIP_2_ alteration in cells. PIP5K also binds to focal adhesion-associated protein talin and produces PIP_2_ from its precursor PPI (36). Our previous overexpression and knockdown/KO studies showed that Pfn1 promotes actin filament (including stress fibers) formation and focal adhesions in various cells including some of those utilized in the present study (*e.g.*, MDA-231 and BT-474) cells (39, 40, 38). Therefore, loss of Pfn1 could also lead to a defect in PIP5K recruitment to the PM partly as a secondary consequence of reduced actin filament/focal adhesion content in cells.

A previous study showed that the basal PIP_2_ hydrolysis activity of PLCγ is inhibited in the presence of Pfn1 initially leading to a speculation that Pfn1 could play a role in negatively regulating PLCγ-mediated PIP_2_ hydrolysis (41). However, a subsequent study from the same group demonstrated that Pfn1 is unable to inhibit the PIP_2_ hydrolysis activity of PLCγ when it is tyrosine-phosphorylated (mimics the activated state of PLCγ in response to growth factor signaling) (42). Since these studies were strictly performed in a test tube setting using synthetic membrane, whether Pfn1 plays a role in growth factor signaling induced PIP_2_ turnover in actual cells remained unclear. We previously showed that Pfn1 depletion does not affect RTK (EGFR, PDGFR) activation when cells are stimulated by appropriate growth factors (EGF, PDGF) (23). In this study, we further show that loss of Pfn1 expression has no effect on EGF-induced PIP_2_ hydrolysis. Although IP_3_ and DAG are the immediate byproducts of PIP_3_ hydrolysis, our assays measured IP1 (a rapid degradation product of IP_3_) as a surrogate measure of IP_3_, an approach that has been widely used (43, 44). Given that Pfn1 depletion leads to reduced steady-state PIP_2_ content yet does not alter EGF-induced IP1 production in cells, theoretically, it can be argued that loss of Pfn1 expression somehow enhances the intrinsic PIP_2_ hydrolysis ability in cells. While this tenet is consistent with Pfn1-induced inhibition of PIP_2_ hydrolysis as previously postulated from biochemical experiments (41), it would require an assumption that the actual PM PIP_2_ content is a limiting factor for the hydrolysis pathway, a scenario that is unlikely given the high abundance of PM PIP_2_. We therefore speculate that Pfn1, if at all, has negligible impact on PLCγ-mediated EGF hydrolysis in cells. This scenario is consistent with our inability to demonstrate Pfn1’s localization in PM upon rapamycin-induced PM recruitment of PIP4K2A-A371E in cells, and suggests that Pfn1’s ability to inhibit the basal PIP_2_ hydrolysis activity of PLCγ in a biochemical assay may be a consequence of Pfn1:PIP_2_ binding in a setting not representative of actual cells. However, we did not investigate the effect of Pfn1 depletion on PIP_2_ hydrolysis by other PLC isoforms (*e.g.*, PLCβ and PLCδ) that are activated by GPCRs. We previously analyzed transcriptomic changes in MDA-231 cells and human telomerase reverse transcriptase-immortalized mouse endothelial cells (MEC) upon stable silencing of Pfn1 expression and acute knockout of the Pfn1 gene, respectively (45, 46). These studies revealed Pfn1-dependent changes in PLCβ1 and/or PLCδ expression and predicted alterations in GPCR and cAMP (activated by GPCR) signaling. While actin filaments clearly can serve as a scaffold to recruit enzymes at the submembranous regions, these structures can also act as barriers to signaling molecules. Disassembly of actin filaments leads to spontaneous activation of PLC through gain of access and phosphorylation by Src family kinases and downstream signaling (such as calcium release) (47, 48, 49). Since loss of Pfn1 reduces actin filament abundance almost universally in all cell types, we cannot yet completely rule out the effect of Pfn1 on PLC activity potentially due to expression changes and/or as a secondary consequence of cytoskeletal changes.

Opposite directionality of Pfn1-dependent changes in PIP_2_ and PIP_3_, taken together with our PIP_2_ hydrolysis data, further suggest that elevated PIP_3_ response upon Pfn1 depletion is not a secondary consequence of either PIP_2_ substrate availability for PI3K-mediated turnover or preferential inhibition of alternate PLCγ-mediated PIP_2_ turnover. How might loss of Pfn1 expression enhance EGF-induced PIP_3_ production in cells? Firstly, our observation is inconsistent with previous reports of Pfn1’s ability to bind to the p85 subunit of PI3K and stimulate the lipid kinase activity of PI3K in biochemical assays (50, 51). A previous study also reported that Pfn1 binds to PTEN (an antagonist of PI3K signaling) and that Pfn1’s interaction protects PTEN from ubiquitination, leading to an increased cellular abundance of PTEN in MDA-231 breast cancer cells when Pfn1 is stably overexpressed (52). Our gene silencing or KO experiments performed in diverse cell types show that acute Pfn1 depletion does not change the expression of either total or phosphorylated form of PTEN, suggesting that Pfn1 is a not limiting factor for the basal PTEN expression. However, we cannot rule out the possibility that PTEN activity at the PM might be sensitive to the loss of Pfn1 expression. Binding to PIP_2_ is critical for PM localization and the catalytic activity of PTEN (53). Given our data showing Pfn1 depletion leads to reduced PM content in cells, it is not out of the realm of possibility that loss of Pfn1 expression reduces PM residence time of PTEN, rather than somehow promoting PI3K activity with a net result of increased PM abundance of PIP_3_. Single-molecule imaging studies in cells expressing endogenously tagged PTEN will be needed in the future to investigate this possibility.

Our PI(3,4)P_2_ biosensor imaging experiments performed in normal HEK-293 cells, together with our previous immunostaining-based findings in MDA-231 breast cancer cells (23) not only strengthen Pfn1-dependent regulation of PI(3,4)P_2_ in a general context, but also demonstrate that loss of Pfn1 expression increases biosynthesis of PI(3,4)P_2_ rather than slowing its degradation. These data are consistent with increased PM availability of PIP_3_ (the precursor PPI for PI(3,4)P_2_ synthesis) as well as enhanced PM recruitment of SHIP2 in Pfn1-depleted condition. Our experimental results tend to rule out a potential competition between Pfn1 and SHIP2 for PM PPI binding as a potential mechanism for enhanced EGF-induced PM recruitment SHIP2 in Pfn1-depleted condition. However, it is important to note that we previously showed that increased PM accumulation of PI(3,4)P_2_ in Pfn1-depleted cells can be reversed by re-expression of WT Pfn1 but not R88L-Pfn1. Since R88L substitution reduces Pfn1’s affinity for PPIs *in vitro*, we initially speculated that Pfn1:PPI interaction as a potential mechanism of negative regulation of PI(3,4)P_2_ (23). While we cannot fully explain this apparent discrepancy at this time, it is possible that structural alteration imposed by R88L mutation has additional consequences on Pfn1’s interaction with other ligands besides PPI interaction. It is known that R88L substitution also reduces Pfn1’s affinity for actin by ∼2-fold (54). There could be at least two possible explanations underlying increased PM recruitment of SHIP2 in the Pfn1 depletion setting. Our PLA data showing evidence for Pfn1-SHIP2 binding and reduction of this interaction by EGF stimulation are consistent with a purely hypothetical model of Pfn1 binding as a restrictive mechanism of PM recruitment of SHIP2. Alternatively, since SHIP2 forms functionally active complex with F-actin and F-ABP filamin (55), Pfn1-dependent changes in the actin cytoskeleton could indirectly impact the subcellular localization of SHIP2. More in-depth studies are clearly needed in the future to either fully support these models or seek alternative mechanisms, which are beyond the scope of the present work.

## Experimental procedures

### Cell culture

HEK-293A cells (Thermo Fisher Scientific R705-07) were cultured in complete media composed of low glucose Dulbecco's modified Eagle's medium (DMEM) (Thermo Fisher Scientific 10567022), 10% heat-inactivated fetal bovine serum (Thermo Fisher Scientific 10438-034), 100 μg/ml penicillin, 100 μg/ml streptomycin (Thermo Fisher Scientific #15140122), and 0.1% chemically defined lipid supplement (Thermo Fisher Scientific #11905031). MDA-231, BT-474, and HeLa cells were cultured in in DMEM supplemented with 10% (v/v) fetal bovine serum, 100 μg/ml penicillin, and 100 μg/ml streptomycin. MCF10A cells were cultured in DMEM:F12 media supplemented with 5% (v/v) horse serum, 100 μg/ml penicillin, 100 μg/ml streptomycin, 2.5 mM L-glutamine, 10 μg/ml insulin, 0.5 μg/ml hydrocortisone, 100 ng/ml cholera toxin, and 20 ng/ml EGF. Generation and culture protocol of human telomerase reverse transcriptase-immortalized MEC with floxed Pfn1 alleles and stable sublines of GFP- and GFP-Pfn1 expressing MDA-231 and BT-474 cells were previously described (38, 40, 56). All cell lines were routinely screened for confirmation of mycoplasma-free status by PCR. Rapamycin (Sigma-Aldrich 80054-244), prepared at a 1 mM stock solution in DMSO, was added to cells at a final concentration of 1 μM. EGF (Thermo Fisher Scientific CB-40052), prepared at 0.1 mg/ml stock solution in deionized water, was added to cells at a final concentration of 100 ng/ml. Digitonin (Sigma-Aldrich 300,410), prepared at a stock concentration of 20 mM, was added to cells at a final concentration of 20 μM.

### Plasmids, siRNAs, and transfection

Majority of our plasmids were engineered on the Clontech pEGFP-C1 and pEGFP-N1 backbones as previously outlined (57). We used mTagBFP2 (58), mCherry (59), EGFP, and iRFP (near infrared fluorescent protein) (60) fluorescent proteins throughout this study. To assemble the plasmids, we employed either standard restriction cloning or NEB HiFi assembly (New England Biolabs #E5520S), followed by verification with Sanger Sequencing. Details of all plasmids are provided in Table S1. For imaging procedures, HEK-293 cells were seeded in 35 mm dishes with 20 mm #1.5 glass bottoms (CellVis #D35-22-1.5-N) in 2 ml complete media. For cell adherence, the glass bottoms were coated with 1 mg/ml ECL (Entactin-Collagen IV-Laminin attachment matrix; Sigma-Aldrich #08-110). For cDNA transfection, cell confluence was allowed to reach 25% and 50% for TIRF and confocal microscopy, respectively. The transfection components included of 0.1 to 1 μg specific plasmid DNA complexed to 3 μg Lipofectamine 2000 (Thermo Fisher Scientific #11668019) in ∼200 μl Opti-MEM (Thermo Fisher Scientific #51985091). The mixture was incubated at room temperature for >5 min before adding to the cells. For siRNA experiments, cells were transfected with siGENOME Pfn1 SMARTpool (Horizon Discovery #M-012003-01-0005) or siGENOME NonTargeting (Horizon Discovery #D-001206-13-05) complexed with DharmaFECT (Horizon Discovery #T-2001-01). Briefly, 50pmol of siRNA was precomplexed with 6.6 μl DharmaFECT in 400 μl serum-free medium (SF-CHIM; detailed in imaging procedure) and then diluted to 2 ml with antibiotic-free complete media before adding to cells. Culture was maintained for up to 72 h with replacement of media performed on the day after transfection. Lipofectamine 2000/3000 and RNAi Max were used for plasmid and siRNA transfection of various cancer cell lines (MDA-231, BT-474, HeLa), respectively, as per manufacturer’s instruction. Select siRNA experiments involved transfection with a second single-target Pfn1 siRNA as described previously (37). Adenovirus-mediated deletion of floxed Pfn1 alleles in MEC was performed as recently described (45).

### Endogenous tagging of cellular proteins

We generated HEK-293A cells endogenously tagged with the split NG2 protein at the Pfn1 and SHIP2 (INPPL1) loci, following a previously published general protocol (57). Briefly, we electroporated a Platinum Cas9 (Thermo Fisher Scientific#B25640)–gRNA complex with a single-stranded HDRT (IDT) into our stably expressing HEK293^NG2-1-10^ cells. The HDRT is composed of 70 bp homology arms to align with the gene of interest, the NG2 11th β-strand insertion sequence, and a flexible linker. HDRT and gRNA sequences for INPPL1 are provided in Table S2. HDRT and gRNA sequences for Pfn1 are from OpenCell database. Following electroporation, we screened cells for fluorescence using our confocal microscope and subsequently used fluorescence-activated cell sorting (University of Pittsburgh Flow Cytometry Core) to isolate and expand NG2-positive cells.

### PIP_2_ hydrolysis assay

We used a commercial kit (catalog # 62IPAPEB) from Cisbio for FRET-based measurement of cellular IP_1_ concentration as a readout of PIP_2_ hydrolysis, following the manufacturer’s protocol. Briefly, cells were serum-starved overnight and stimulated with 100 ng/ml EGF for 30 min (unstimulated cells served as the control). Cell lysates were incubated with IP_1_-Tb-cryptate antibody (donor) and IP_1_-d2 reagent (acceptor). In this assay, IP_1_ produced by cells compete with IP_1_-d2 for binding to the anti-IP_1_-Tb-Cryptate antibody, reducing the FRET signal which was read on an HTRF compatible plate reader at 665 nm wavelength setting. Based on EC50 dose-response curve of a serial dilution of IP1 standard stock, cellular IP1 concentration of six replicates/group over three experiments were quantified.

### PPI immunostaining

Cells were plated on the wells of a 8-well glass-bottom chamber slides coated with collagen. For PIP_3_ staining, cells were serum-starved overnight and stimulated with 100 ng/ml for 5 min. Cells were fixed with 3.7% (w/v) paraformaldehyde/0.1% glutaraldehyde for 1 h at 37 °C and permeabilized with 0.15 mg/ml saponin solution. These solutions were prepared in fixation buffer (5 mM KCl, 137 mM NaCl, 4 mM NaHCO_3_, 0.4 mM KH_2_PO_4_, 1.1 mM Na_2_HPO_4_, 2 mM MgCl_2_, 5 mM piperazine-N,N′bis(2-ethanesulfonic acid), pH 7.2, 2 mM EGTA and 5.5 mM glucose. After blocking with 5% (w/v) BSA for 45 min, cells were incubated with primary antibodies (mouse monoclonal anti-PIP_3_, anti-PIP_2_, and anti-PI(4)P [source: Echelon Biosciences] at a 1:100 dilution) for 1 h at room temperature. After washing 3 to 4 times in PBS, cells were incubated with appropriate secondary antibodies for 45 min and washed 3 to 4 times in PBS before mounting (6 mg/ml propyl gallate prepared in 50% (v/v) glycerol/PBS) and acquisition of fluorescence images. Fluorescence images were taken with either 20× or 60× objective on Olympus wide-field and Zeiss confocal microscopes. All images were background subtracted using ImageJ (https://imagej.net/ij/) software for quantitative fluorescence intensity analyses.

### PIP_2_ dot blot assays

Cells grown to ∼80% culture confluency was treated with prechilled 0.5 M trichloroacetic acid was added and scraped. The mixture was transferred to a 15 ml conical tube and centrifuged at 3000 rpm for 7 min at 4 °C. After discarding the supernatant, cell pellet was washed 2× by 5% (v/v) trichloroacetic acid/1 mM EDTA and centrifuged at 3000 rpm for 5 min. After removing the supernatant, neutral lipids were extracted by adding 2:1 MeOH:CHCl_3_, vortexing for 10 min, and centrifuging at 3000 rpm for 5 min. After discarding the supernatant, the acidic lipids were then isolated by adding 80:40:1 MeOH:CHCl_3_:12N HCl solution and vortexing the cell pellet for 30 min. After centrifuging, the supernatant was transferred to a new conical tube and pure CHCl_3_ diluted in 0.1 N HCl was added to split the organic and aqueous phases. A positive displacement pipette was used to transfer the organic phase into fresh Eppendorf tubes and the samples were vacuum dried overnight. The dried samples were stored at −20 °C until the time of use. Lipids spotted on nitrocellulose membrane were blocked with 5% milk in Tris-buffered saline with 0.1% tween and incubated with 1:200 mouse anti-PIP_2_ antibody (Echelon, Z-P045) overnight at 4 °C. The membrane was then incubated with 1:1000 horseradish peroxidase-conjugated anti-mouse immunoglobulin M (IgM) (BD Pharmingen, 554002) for 1 h at room temperature before washing 3× with Tris-buffered saline with 0.1% tween and chemiluminescence imaging.

### Proximity ligation assay

PLA was performed using the Duolink kit with antimouse plus and antirabbit minus probes (Sigma, DUO9210). Cells cultured on coverslips were washed with Dulbecco's PBS, fixed with 3.7% (v/v) formaldehyde/PBS for 15 min, permeabilized with 0.5% (v/v) Triton X-100/PBS for 5 min, and then blocked with 10% (v/v) goat serum/PBS for 1 h at room temperature. *In situ* proximity ligation was performed using two primary antibodies of different species targeting either Pfn1 (Abcam, 1:200, ab242369, mouse) or SHIP2 (CST, 1:100, 2839, rabbit), both for 1 h at room temperature in 10% goat serum, and a pair of oligonucleotide-labeled secondary antibodies targeting each primary antibody from the Duolink kit according to the protocol of the manufacturer. PLA probe hybridization, ligation, amplification, and detection were performed according to the protocol of the manufacturer. To quantify PLA spots, images (acquired at an emission wavelength of 624 nm with a ×60 oil immersion objective (N.A. = 1.4) on an Olympus IX-83 inverted microscope) were first background-subtracted using the mean fluorescence intensity of cells from the negative control group. A mask was then created from the images of interest, and the analyze particles option was used to count the number of spots in each cell from a field of view.

### Microscopy

Microscopy experiments (pertinent to Figs. 1, 5–7) were conducted with specific imaging media. We used SF-CHIM media, composed of Fluorobrite DMEM (Life Technologies A1896702), 25 mM Hepes (pH 7.4), and 0.01% chemically defined lipid supplement. For time-course experiments, the media additionally contained 0.1% (w/v) BSA. For cell permeabilization studies, cells were washed with KHME solution composed of 110 mM potassium acetate (K), 20 mM Hepes (H), 2 mM MgCl2 (M), and 0.1 mM EGTA (E), just before imaging.

#### Confocal microscopy

We performed confocal microscopy on a Nikon TiE A1R platform set to resonant mode. To excite the fluorophores on our protein of interest, we used a dual-fiber coupled LUN-V laser launch. The launch contained four laser lines, 405 nm to excite BFP; 488 nm to excite EGFP and NG2; 561 nm to excite mCherry; and 640 nm to excite iRFP. To collect emission following fluorophore excitation, green (505–550 nm), yellow-orange (570–620 nm), and far-red (650–850 nm) filters were used. A 1.45 Na plan apochromatic oil immersion objective from Nikon was used to obtain the images.

#### TIRF microscopy

To perform TIRF microscopy, we used a TiE inverted microscope with a fixed motorized TIRF illuminator by Nikon. Protein fluorophores were excited using our Oxxius L4C laser combiner. The combiner contained four laser lines, 405, 488, 561, and 640 nm. We imaged with a 100 to 200 ms frame delay, wide-field fluorescence modality, 2 × 2 pixel binning, Sutter Lambda 10 to 2 Filter Changer, Zyla 5.5 cCMOS camera, and in standard and ultraquiet scan modes. BFP fluorophores (blue; 405 nm channel) were imaged with the ZET405/561 dual-pass 420 to 480 nm Chroma filter; mCherry fluorophores (yellow/orange; 561 nm channel) were imaged with the TRITC (Em) Chroma filter; the EGFP/NG fluorophores (green; 488 nm channel) were imaged with a FITC (Em) Chroma filter; the iRFP fluorophores (infrared; 640 nm) were imaged with the ZET488/640 dual-pass 505 to 550 nm and 650 to 850 nm Chroma filter.

#### Image analysis

Images produced with the confocal and TIRF microscope assemblies were saved as Nikon nd2 files and analyzed with the Fiji (https://fiji.sc) open-access analysis package. The nd2 files were imported with the LOCI Bioformats importer and specialized macros were used to produce experiment-specific analysis. To quantify the change in fluorescence intensity in different compartments (for studies shown in Fig. 1), regions of interest (ROIs) were traced around each cell's outline, and the average pixel intensity in these areas was generated. This was then normalized to the specific compartments (PM) binary mask (61), measuring the change in fluorescence intensity at the PM over time, as further outlined in (57). Single-molecule density was measured within set 100 μm^2^ ROIs, our specific macro resolved single molecules to approximately 20 nm and counted the molecules at the cell surface over time (for studies shown in Fig. 5). For overexpressed construct analysis the pixel intensity within a cell ROI was averaged to produce F_t_, this was baseline-corrected to the average pixel intensity before stimulation (F_pre_), producing a normalized ratio analysis (F_t_/F_pre_) (for studies shown in Figs. 6 and 7).

### Protein extraction/immunoblotting

Total cell lysate was collected using a modified RIPA buffer (25 mM Tris–HCl, pH 7.5, 150 mM NaCl, 1% (v/v) Nonidet P-40, 5% (v/v) glycerol), 1 mM EDTA, 50 mM NaF, 1 mM sodium pervanadate, along with 6× sample buffer with SDS diluted to 2% in final buffer. Total cell lysate was run on an SDS-PAGE and immunoblotted using various antibodies (see Table S3 for details of antibody information).

### Statistics

All statistical tests were performed using GraphPad Prism 9 software (https://www.graphpad.com). ANOVA (for more than two groups) and Student’s *t* test (for two groups) were performed for comparing means between various experimental groups. A *p*-value less than 0.05 was considered to be statistically significant.

## Data availability

All data are included in the manuscript and supplementary information.

## Supporting information

The article contains supporting information.

## Conflict of interest

The authors declare that they have no conflict of interest with the contents of this article.
