# Supplementary material for: Actin-binding protein profilin1 is an important determinant of cellular phosphoinositide control
Source: J Biol Chem. 2023 Dec 21;300(1):105583. doi: 10.1016/j.jbc.2023.105583 (PMC10826164; doi:10.1016/j.jbc.2023.105583)
Supplement: Supporting information [file mmc1.pdf]

Ricci et al. Supporting Information

Ricci et al. Figure S1

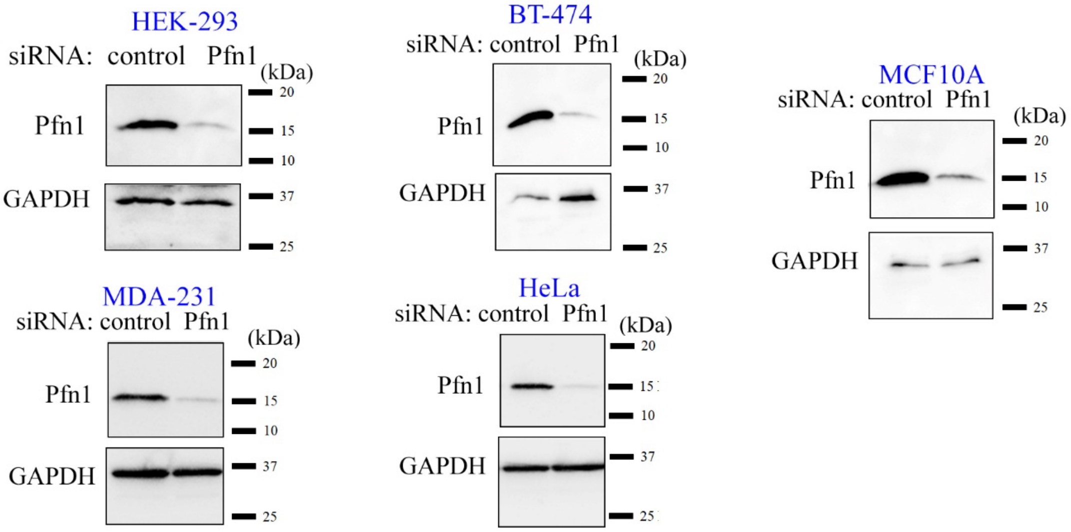

Fig S1: Validation of siRNA-mediated Pfn1 knockdown: Pfn1 Immunoblot of total cell lysates prepared from the indicated cell lines transiently transfected with either smart-pool Pfn1 siRNA or smart-pool non-targeting control siRNA (GAPDH blots serve as the loading control).

Ricci et al. Fig S2

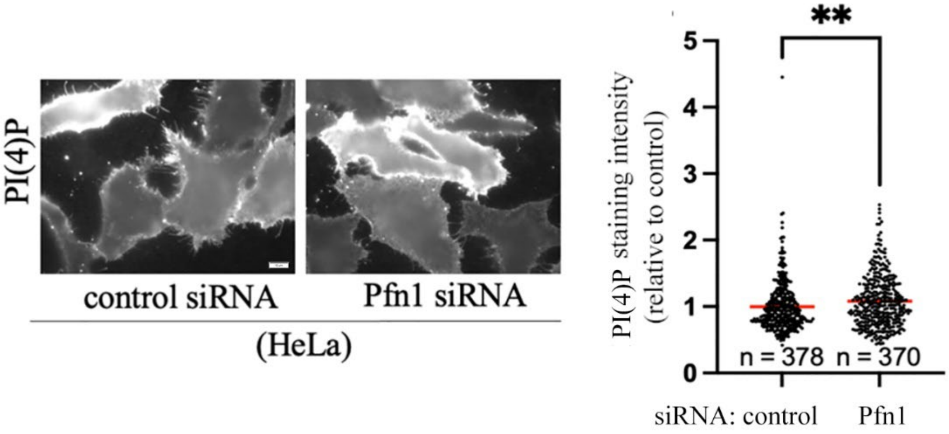

Fig S2: **Silencing Pfn1 expression has negligible impact on PM PI(4)P content in cells**. Representative images and quantification ('n' indicates cell number pooled from two experiments) of staining intensity of PI(4)P with or without Pfn1 knockdown in HeLa cells (**: p<0.01; scale bar – 10 µm).

Ricci et al. Fig S3

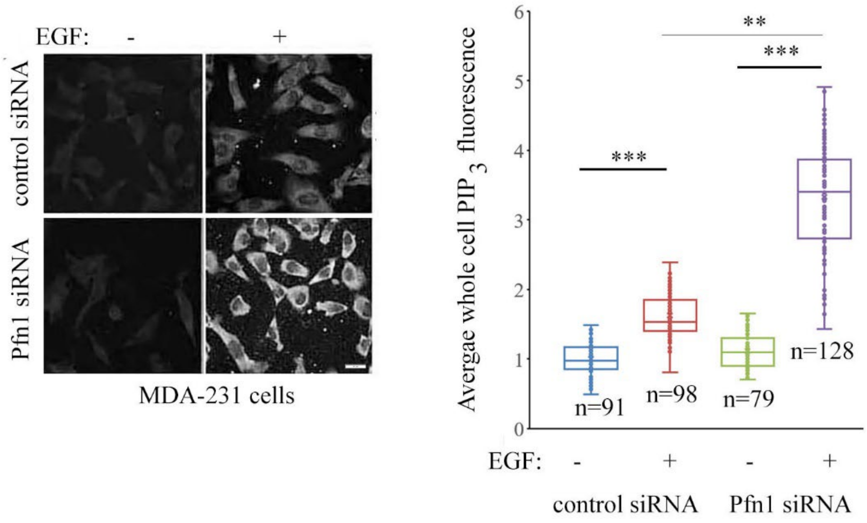

Fig S3: **Additional evidence for Pfn1-dependent changes in EGF-induced PIP_3 accumulation in cells**. Representative immunostaining images and quantification ('n' indicates number of analyzed cells pooled from two experiments) of PIP_3 in control vs Pfn1-silenced MDA-231 cells before and 5 min after EGF stimulation. Note that fixation protocol employed in this experiment was different from that adopted in experiments with HeLa cells as shown in **Fig 4** (**: $p<0.01$; ***: $p<0.001$; scale bar – 50 μm).

Ricci et al. Fig S4

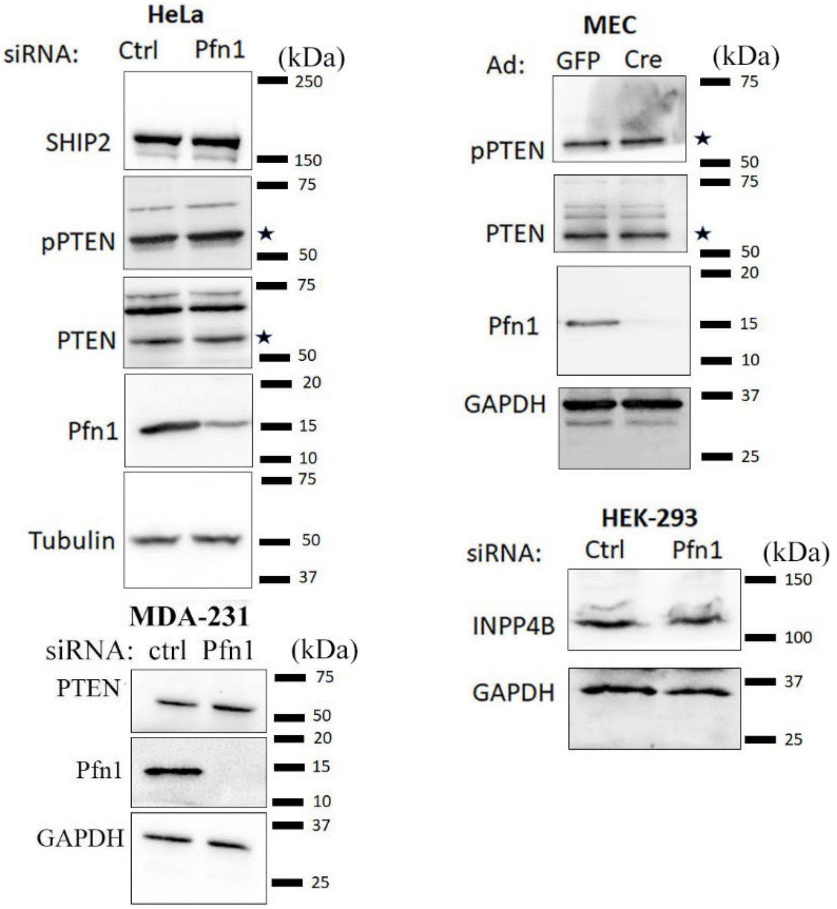

Fig S4: **Loss of Pfn1 expression does not affect the expressions of lipid phosphatases responsible for generation and degradation of PI(3,4)P_2.** Immunoblot analyses for SHIP2, PTEN, pPTEN and INPP4B in the indicated cell lines with or without forced suppression of Pfn1 expression either by siRNA transfection or adenovirus (Ad)-mediated knockout of floxed Pfn1 alleles (as shown in immortalized mouse endothelial cells [MEC]; Ad-GFP: control). GAPDH and tubulin blots serve as the loading controls. For certain PTEN and pPTEN blots, 55 kDa PTEN- and pPTEN-specific bands are marked by asterisks to distinguish those from occasional non-specific higher molecular bands recognized by the respective antibodies in a cell line-specific manner.

Table S1: Details of plasmids used in the study

Plasmid	Backbone	Insert	References
NES-NG-C1-TAPP1-cPHx3	pNES-EGFP-C1	*X. laevis* map2k1.L(32-44):EGFP:PLEKHA1(169-329):GGSGGSGG: PLEKHA1(169-329): GGSGGSGG: PLEKHA1(169-329)	Goulden et al 2019
Tag2BFP-Tubby(c)-R332H	pTagBFP2	*Mus muscularis*:Tubby(243-505):Tag2BFP	Quinn et al 2008
MCherry-C1	pmCherry-C1	mCherry	Shaner et al., 2004
NeonGreen-C1	pmNeonGreen-C1	mNeonGreen	Shaner et al., 2013
pNES-iRFP-C1	piRFP-C1	*X. laevis* map2k1.L(32-44):iRFP	Goulden et al 2019
mCherry-C1-FKBPflex-SHIP2	mCherry-C1	mCherry: *FKBP1A*(3-108):[GGSA]4GG:INPPL1	This Study
Lyn11-FRB-iRFP	piRFP-N1	*LYN*(1-11):*MTOR*(2021–2113):iRFP	Hammond et al. 2014
mCherry-C1-FKBP-iSH2	pmCherry-C1	mCherry:AAAGAGGAA:*FKBP1A*(3-108): [GGSA]4GG:Mus musculus *Pik3r1*(159-349)	Suh et al., 2006
mCherry-C1-FKBPflex-HsPIP4K2A-A371E	pmCherry-C1	mCherry:*FKBP1A*(3-108): [GGSA]4GG:PIP4K2A	This Study
mCherry-C1-FKBP-INPP5E	pmCherry-C1	mCherry:*FKBP1A*(3-108):[GGSA]4GG:INPP5E(214-644)	Hammond et al 2014
mCherry-C1-PJ	pmCherry-C1	mCherry:*FKBP1A*(3-108): [GGSA]4GG:Sac1(2-517):INPP5E(214-644)	Hammond et al 2012
mCherry-C1-FKBP-INPP5E-D556A	pmCherry-C1	mCherry:*FKBP1A*(3-108):[GGSA]4GG:INPP5E(214-644)	Hammond et al 2014
mCherry-C1-FKBPflex-PTEN	pmCherry-C1	mCherry:*FKBP1A*(3-108):[GGSA]4GG:PTEN	Goulden et al 2019

References

Goulden BD, Pacheco J, Dull A, Zewe JP, Deiters A, Hammond GRV. A high-avidity biosensor reveals plasma membrane PI(3,4)P2 is predominantly a class I PI3K signaling product. J Cell Biol. 2019 Mar 4;218(3):1066-1079. doi: 10.1083/jcb.201809026. Epub 2018 Dec 27. PMID: 30591513; PMCID: PMC6400549.

Quinn, K.V., Behe, P. and Tinker, A. (2008), Monitoring changes in membrane phosphatidylinositol 4,5-bisphosphate in living cells using a domain from the transcription factor tubby. The Journal of Physiology, 586: 2855-2871. https://doi.org/10.1113/jphysiol.2008.153791

Shaner NC, Campbell RE, Steinbach PA, Giepmans BN, Palmer AE, Tsien RY. Improved monomeric red, orange and yellow fluorescent proteins derived from Discosoma sp. red fluorescent protein. Nat Biotechnol. 2004 Dec;22(12):1567-72. doi: 10.1038/nbt1037. Epub 2004 Nov 21. PMID: 15558047.

Shaner, N., Lambert, G., Chammas, A. *et al.* A bright monomeric green fluorescent protein derived from *Branchiostoma lanceolatum*. *Nat Methods* 10, 407–409 (2013). https://doi.org/10.1038/nmeth.2413

Hammond GR, Machner MP, Balla T. A novel probe for phosphatidylinositol 4-phosphate reveals multiple pools beyond the Golgi. J Cell Biol. 2014 Apr 14;205(1):113-26. doi: 10.1083/jcb.201312072. Epub 2014 Apr 7. PMID: 24711504; PMCID: PMC3987136.

Hammond GR, Fischer MJ, Anderson KE, Holdich J, Koteci A, Balla T, Irvine RF. PI4P and PI(4,5)P2 are essential but independent lipid determinants of membrane identity. Science. 2012 Aug 10;337(6095):727-30. doi: 10.1126/science.1222483. Epub 2012 Jun 21. PMID: 22722250; PMCID: PMC3646512.

Suh B.-C., Inoue T., Meyer T., and Hille B.. 2006. Rapid chemically induced changes of PtdIns(4,5)P2 gate KCNQ ion channels. *Science.* 314:1454–1457. 10.1126/science.1131163

Table S2: HDRT and crRNA sequences of INPPL1 and Pfn1 knock-in constructs

**INPPL1-NG11 HDRT**	CTTGGAGGAGGCTGGGGTGCAGGACCCGGCTCACAAGCGCCTCCTTCTGGACACCCTGCAGCTCAGCAAGGGTGGCGGCACCGAGCTCAACTTCAAGGAGTGGCAAAAGGCCTTTACCGATATGATGTGATAGCGGAGGCACCACGAAGCTGTGAACTCAGAGCCCCTCCCTGCTACCAAGGCCCAGCTATGGCCCC
**INPPL1 crRNA**	TGCAGCTCAGCAAGTGATAG
**NG11 - Pfn1 HDRT**	CGTCCTGACAGGTCCCGTCCGCCATGAGGTTGTCGATGTAGGCGTTCCACCCCGCACCACTTCCTGGACCTTGAAACAAAACTTCCAATCCGCCACCCATCATATCGGTAAAGGCCTTTTGCCACTCCTTGAAGTTGAGCTCGGTCATGGCGCTGCTACTGGGGCTGCTCTCGGCGCTGCTGCTGGGGCCGCGGACTGGG
**Pfn1_crRNA**	GTAGGCGTTCCACCCGGCCA

Table S3. Details of primary antibodies used for immunoblot experiments

**Antibody**	**Species**	**Type**	**Dilution**	**Source**
GAPDH	rabbit	monoclonal	(1:2000)	Invitrogen
INPP4B	rabbit	polyclonal	(1:1000)	AbClonal
PTEN	rabbit	monoclonal	(1:1000)	Cell Signaling Technologies
pPTEN ser380/Thr382/383	rabbit	monoclonal	(1:1000)	Cell Signaling Technologies
Pfn1	rabbit	monoclonal	(1:1500)	Abcam
Tubulin	mouse	monoclonal	(1:1000)	ThermoFisher
SHIP2	rabbit	monoclonal	(1:1000)	Cell Signaling Technologies
